# Manipulating the Rapid Consolidation Periods in a Learning Task Affects General Skills More than Statistical Learning and Changes the Dynamics of Learning

**DOI:** 10.1523/ENEURO.0228-22.2022

**Published:** 2023-02-23

**Authors:** Laura Szücs-Bencze, Lison Fanuel, Nikoletta Szabó, Romain Quentin, Dezso Nemeth, Teodóra Vékony

**Affiliations:** 1 Department of Neurology, University of Szeged, H-6725, Szeged, Hungary; 2 Université Claude Bernard Lyon 1, CNRS, INSERM, Centre de Recherche en Neurosciences de Lyon CRNL U1028 UMR5292, 95 Boulevard Pinel, F-69500, Bron, France; 3 Institute of Psychology, ELTE Eötvös Loránd University, H-1064, Budapest, Hungary; 4 Brain, Memory and Language Research Group, Institute of Cognitive Neuroscience and Psychology, Research Centre for Natural Sciences, H–1117, Budapest, Hungary

**Keywords:** general skill learning, rapid consolidation, statistical learning

## Abstract

Memory consolidation processes have traditionally been investigated from the perspective of hours or days. However, recent developments in memory research have shown that memory consolidation processes could occur even within seconds, possibly because of the neural replay of just practiced memory traces during short breaks. Here, we investigate this rapid form of consolidation during statistical learning. We aim to answer (1) whether this rapid consolidation occurs in implicit statistical learning and general skill learning, and (2) whether the duration of rest periods affects these two learning types differently. Human participants performed a widely used statistical learning task—the alternating serial reaction time (ASRT) task—that enables us to measure implicit statistical and general skill learning separately. The ASRT task consisted of 25 learning blocks with a rest period between the blocks. In a between-subjects design, the length of the rest periods was fixed at 15 or 30 s, or the participants could control the length themselves. We found that the duration of rest periods does not affect the amount of statistical knowledge acquired but does change the dynamics of learning. Shorter rest periods led to better learning during the learning blocks, whereas longer rest periods promoted learning also in the between-block rest periods, possibly because of the higher amount of replay. Moreover, we found weaker general skill learning in the self-paced group than in the fixed rest period groups. These results suggest that distinct learning processes are differently affected by the duration of short rest periods.

## Significance Statement

Results of this study suggest that short rest periods affect general skill learning and the dynamics of statistical learning. Shorter rest periods could lead to online learning, while longer rest periods promote offline improvement. Our results can be explained by the different number of neural replays during the different lengths of short rest periods.

## Introduction

Learning is the process of gaining knowledge or skills by studying, practicing, or experiencing events repeatedly. The development of knowledge is not limited to the duration of the practice, as it continues to develop between training sessions, either during awake or sleep periods. This phenomenon is known as memory consolidation (Robertson et al., 2004b). Consolidation was previously thought to occur during an extended period, from hours to days (Squire et al., 2015). Recent studies suggest that memory consolidation can occur within shorter periods, even in seconds (Bönstrup et al., 2019). It has been suggested that this phenomenon is because of the neural replay of just practiced memory traces during short breaks (Buch et al., 2021). However, previous research has investigated the effect of short rest periods—when replays occur—on learning only with one predetermined fixed rest period (Du et al., 2016; Bönstrup et al., 2019) or multiple self-paced rest periods (Quentin et al., 2021; Fanuel et al., 2022). Their results do not allow us to determine the causal role of short rests in the learning process, or whether more replay leads to better learning performance. To fill this gap, in the present study, we manipulated the duration of rest periods—indirectly the possible amount of replay—to test whether rest durations affect differently (1) the general speedup on a statistical learning task independent of the statistical probabilities in the task (general skill learning) and (2) the learning of statistical probabilities (statistical learning).

Rest periods inserted in a learning process may facilitate the acquisition of new skills (Walker et al., 2002). In the study of Bönstrup et al. (2019), the performance on an explicit motor skill learning task improved during short, 10 s rest periods. In their study, frontoparietal β oscillatory activity during rest periods was associated with learning gains from rapid consolidation. A reanalysis of these data suggested that such rapid consolidation is driven by the replay of just practiced memory traces during short breaks (Buch et al., 2021). As the awareness of learning determines how knowledge acquisition gains from offline periods (Robertson et al., 2004a, b), this rapid consolidation may manifest differently or even be absent during implicit learning of statistical regularities. Implicit statistical learning implies the process of unintentional acquisition of probabilistic regularities embedded in the environment (Cleeremans and Jimenez, 1998; Howard et al., 2004). So far, two studies have examined the rapid consolidation in implicit statistical learning. On one hand, they have found that implicit acquisition of statistical knowledge does not improve during short breaks but deteriorates, and this effect is not associated with the length of rest periods (Fanuel et al., 2022). On the other hand, statistical learning was found to develop during practice (online), indicating that it benefits from evidence accumulation during practice and the information learned does not consolidate during short rest periods (Quentin et al., 2021).

**Figure 1.**
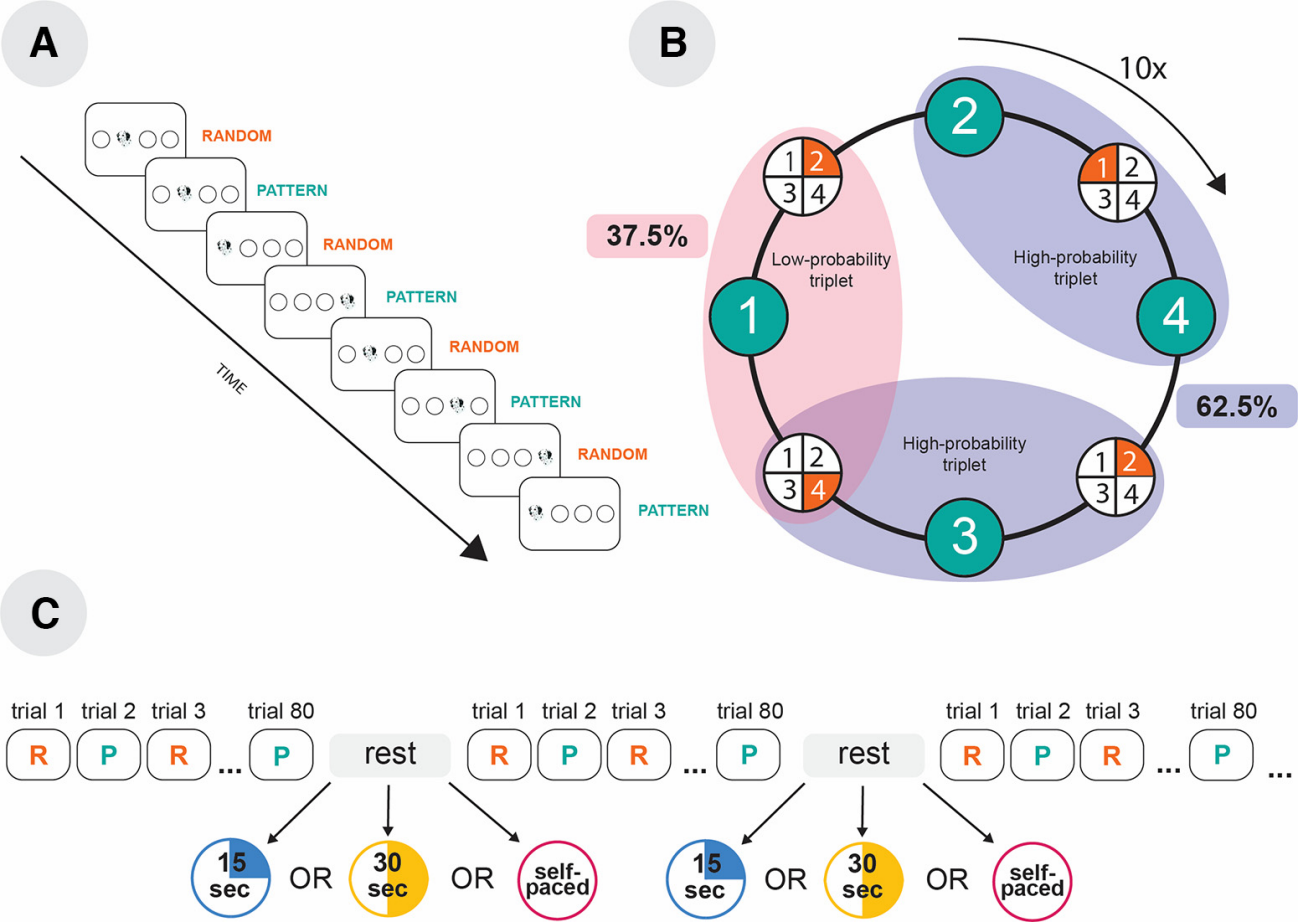
The ASRT task and the study design. ***A***, The temporal progress of the task. A drawing of the head of a dog appeared as a target stimulus in one of four horizontally arranged locations. The stimuli followed a probabilistic sequence, where every other trial was a part of a four-element fixed sequence (pattern elements) interspersed with random elements. ***B***, The formation of triplets in the task. In the eight-element probabilistic sequence, pattern (green) and random (orange) trials alternated. Numbers 1–4 represent the location of the four circles from left to right. Every trial was categorized as the third element of three consecutive trials (i.e., a triplet). Because of the probabilistic sequence structure, some triplets appeared with higher probability (high-probability triplets) than others (low-probability triplets). The ratio of high-probability triplets was higher (62.5% of all trials) than that of low-probability triplets (37.5% of all trials). The eight-element alternating sequence was repeated 10 times in a learning block. ***C***, Study design. Each block contained 80 trials. The between-blocks rest period was 30 s (30 s group), 15 s (15 s group), or a self-paced duration (self-paced group).

Based on previous results, implicit statistical learning occurs only online and does not benefit from rapid consolidation. However, previous studies have a crucial limitation: the length of the rest periods was not controlled experimentally. In the present study, to grasp a causal relationship between the length of breaks and the learning performance, we varied the rest period duration between participants. We aimed to test whether shorter and longer rest durations affect the performance of implicit statistical learning (i.e., the learning of probabilistic regularities) and general skill learning (i.e., the general speedup on a learning task independent of the statistical probabilities). To tackle this question, we used the alternating serial reaction time (ASRT; Fig. 1*A*,*B*) task (Howard et al., 2004), which enables us to measure these two aspects of learning separately. Healthy adults performed 25 blocks of the ASRT task (one block = 80 trials) and were offered to rest after each block. The rest period was (1) a shorter 15 s break, (2) a 30 s break, or (3) a self-paced duration (Fig. 1*C*). As rapid consolidation is related to neural replay (Buch et al., 2021), we expected that the extended rest periods would benefit implicit statistical learning more than the shorter rest periods because of the higher amounts of replays. Moreover, we aimed to test whether there is dissociation in the temporal dynamics of general skill learning and statistical learning regarding online and offline changes and how it is affected by the length of the rest periods.

## Materials and Methods

### Participants

There were 361 participants in this preregistered, online study (https://osf.io/pfy7r). Participants were university students and gained course credits for their participation. Following careful quality control of participant data (see below in the section Quality control of data), the final sample consisted of 268 participants (age: mean = 21.46 years; SD = 2.20 years; 77.61% female, 22.39% male). Participants were randomly divided into three groups (15 s break, 30 s break, and self-paced). Participants in the three groups did not differ in age, education, sex, handedness, or working memory performance (Table 1). All participants had normal or corrected-to-normal vision, and none of them reported a history of any neurologic or psychiatric condition. Participants provided informed consent, and all study procedures were approved by the Research Ethics Committee of the Eötvös Loránd University (Budapest, Hungary) and was conducted in accordance with the Declaration of Helsinki.

**Table 1:** Descriptive statistics of the three experimental groups

	Self-paced group (*n* = 88)	15 s group (*n* = 90)	30 s group (*n* = 90)	Comparison
Age (years)	21.89 ± 2.07	21.24 ± 2.23	21.27 ± 2.26	*p *=* *0.09, BF_01_ = 2.76
Education (sec/BA/MA)	68/17/3	67/22/1	73/16/1	*p *=* *0.56, BF_01_ = 16.37
Gender (M/F)	18/70	20/70	22/68	*p *=* *0.82, BF_01_ = 16.43
Handedness (l/r/a)	8/80/0	8/80/2	10/78/2	*p = *0.68, BF_01_ = 10.02
2-back task (*d*′)	1.57 ± 0.95	1.49 ± 0.87	1.52 ± 0.89	*p *=* *0.82, BF_01_ = 21.03

Mean and SD values for age and 2-back task are presented. For education (sec, secondary education or lower; BA, bachelor’s level or equivalent; MA, master’s level or equivalent), gender (M, male; F, female), and handedness (l, left, r, right, a, ambidextrous), case numbers are presented. To compare age and 2-back task scores, one-way ANOVAs were conducted. To compare education, gender, and handedness ratios, χ^2^ tests were conducted.

### Alternating serial reaction time task

We used the ASRT task to measure implicit statistical and general skill learning separately. The ASRT task was programmed in JavaScript using the jsPsych framework [de Leeuw, 2015; code is openly available on GitHub: https://github.com/vekteo/ASRT_rapid_consolidation (see also https://doi.org/10.5281/zenodo.7124730)]. During the ASRT task, a visual stimulus appeared on the screen (a drawing of the head of a dog) in one of four horizontal locations. Participants must have indicated the location of the target stimulus by pressing the corresponding key on the keyboard (from left to right, the S, F, J, and L keys on a computer keyboard). Participants were instructed to use their left and right middle and index fingers to respond to the targets. Unknown to the participants, the stimuli followed a probabilistic eight-element sequence, where pattern and random elements alternated with each other (e.g., 2 - r - 4 - r - 3 -r - 1 -r, where *r* indicates a random location, and the numbers represent the predetermined positions from left to right). Each participant was randomly assigned to 1 of 24 possible sequences (as the permutation of the 8-element sequence structure allowed 24 different sequences) and then was exposed to that one sequence throughout the task. Because of the probabilistic sequence structure, some runs of three consecutive stimuli (triplet) appeared with higher probability (high-probability triplets) because the third element of such triplets can be predicted by the first trial with a greater probability (62.5% of all trials) compared with third elements of other triplets that can be predicted by the first trial with a lower probability (low-probability triplets, 37.5% of all trials). We can sort every item according to whether they are the third element of a high-probability or a low-probability triplet. Statistical learning was defined as the increase of reaction time (RT) difference between trials that were the third element of a high-probability triplet or low-probability triplet. General skill learning was defined as the overall speeding up on the task (i.e., smaller RTs in later blocks), regardless of the probability of the occurrence of items.

### Process dissociation procedures task

To determine whether the learning of statistical regularities occurred implicitly, we administered a task based on the process dissociation procedure (PDP; Jacoby, 1991), which is a widely used method to disentangle the explicit–implicit processes in memory tasks (Destrebecqz and Cleeremans, 2001; Destrebecqz et al., 2005; Jiménez et al., 2006; Fu et al., 2010). In the first part of the task, we asked participants to try to create a sequence with the help of the same four response keys as used in the ASRT task (inclusion instruction). After that, we asked participants to generate new sequences that differed from the learned sequence (exclusion condition). Both parts consisted of four runs, and each run lasted up to 24 button presses, equivalent to three rounds of the eight-element alternating sequence (Kóbor et al., 2017; Horváth et al., 2020). The runs where >50% of participants’ key presses were either repetitions or trills were removed from the analysis. As a result, seven participants from the self-paced group, three participants from the 15 s group, and three participants from the 30 s group were removed entirely from the analysis, as their answers only contained trills and repetitions in the exclusion condition.

We assessed the implicitness of the participants’ knowledge by calculating the ratio of high-probability triplets in the sequence of responses. The chance level of generating high-probability triplets was considered 25% because, after two consecutive button presses, the chance for the third button press to form a high-probability triplet with the two preceding button presses is 1/4 = 25%. We also compared the percentages of the high-probability triplets across conditions (inclusion and exclusion condition) and groups (self-paced, 15 s, 30 s) (see also https://doi.org/10.5281/zenodo.7253644).

### Procedure

We used the Gorilla Experiment Builder (https://www.gorilla.sc) to host our experiment (Anwyl-Irvine et al., 2020), which allows accurate stimulus and response timing in online experiments (Anwyl-Irvine et al., 2021). Data were collected between April 13, 2021, and October 31, 2021 (experiment material is available on Gorilla Open Materials, https://app.gorilla.sc/openmaterials/397611). Participants were randomly assigned to one of three versions of the task, which differed only in the duration of between-block rest periods. The between-block rest periods were either (1) 15 s breaks, (2) 30 s breaks, or (3) self-paced (i.e., participants were allowed to continue the task with the next block whenever they were ready). The participants performed two practice blocks, then continued with 25 learning blocks, which took ∼25 min to complete. Each block consisted of 80 trials, corresponding to the eight-element sequence repeated 10 times. Accuracy and RT were recorded for each trial. After accomplishing the ASRT task we tested the participants’ awareness of the hidden structure with a short questionnaire and a task based on the process dissociation procedure, which enables us to differentiate explicit and implicit processes in memory tasks (Jacoby, 1991). Finally, they performed 0-back and 2-back tasks (Kirchner, 1958) to assess their working memory capacity (see https://doi.org/10.5281/zenodo.7100178). Data are available on OSF (https://osf.io/ukbfz/).

### Quality control of data

We have set up exclusion criteria before the analysis of the data. Participants were deemed unreliable and were excluded if (1) they did not reach 80% accuracy on the ASRT task (34 participants were excluded for this reason), as in laboratory experiments the general accuracy on the ASRT task is typically >90% (Janacsek et al., 2012); or (2) they performed the 0-back task with <60% accuracy (8 participants), (3) did not complete the *n*-back tasks correctly (i.e., did not press response keys during the task; 16 participants), or (4) had quit the experiment and restarted later (4 participants); or (5) indicated that they had already taken part in an ASRT experiment (8 participants); and (6) had not started blocks on time after the rest period expired (21 participants). We fixed this limit at 1500 ms after the end of the rest period in at least five blocks, and we also excluded the participants whose average RT for the first trials of blocks was >1000 ms (9 participants in the 15 s group; 12 participants in the 30 s group). In addition to the participants excluded according to the predetermined exclusion criteria, as the age range was wide and unequal among the groups, outlying participants (age >35 years) were also excluded (11 participants).

### Quantification of statistical learning and general skill

Inaccurate responses, trills (e.g., 1–2–1) and repetitions (e.g., 1–1–1), and trials with an RT of >1000 ms were excluded from the analysis. There was a total of 535,994 trials, from which a total of 49,927 (9.31%) incorrect trials were excluded. Regarding triplets, 48,715 (9.09%) were trills (e.g., 1-2-1), and 16,302 (3.04%) were repetitions (e.g., 1-1-1). Furthermore, there were 1304 trials (0.24% of all trials) with RTs >1000 ms. As there were overlaps between the trials with different exclusion criteria (e.g., from the 48,715 trills, 7544 were also incorrect trials), the total number of excluded trials is not the sum of the numbers of the different types of excluded trials. We excluded a total amount of 108,344 trials (20.22% of all trials).

To facilitate data processing and filter out noise, the blocks of ASRT were organized into units of five consecutive blocks (Bennett et al., 2007; Barnes et al., 2008; Nemeth et al., 2010), for which we calculated statistical learning and general skill learning scores. Each task trial was categorized as the third element of a low-probability or a high-probability triplet (except the first two trials of each block that could not have been categorized as the third element of a high-probability or low-probability triplet). To measure the degree of implicit statistical learning, we calculated a statistical learning score by subtracting the median RT of the high-probability triplets from the median RT of the low-probability triplets. Then, to control the difference in base RTs between groups, we divided this learning score by the mean RT (standardized statistical learning scores). To measure general skill learning, the median RTs of each unit of five blocks were calculated regardless of the probability of the occurrence of items.

### Quantification of online and offline changes

Further scores were calculated to compare the online and offline general skill learning and statistical learning changes. Each block of 80 trials was divided into five bins (each containing 16 consecutive trials). For each bin, we calculated the difference between high-probability and low-probability triplets, resulting in a single learning score for each bin for each block. To calculate the online change in statistical learning, we subtracted the learning score of the first bin from that of the last bin of the same block (the change from the beginning to the end of the block). Twenty-five scores were obtained corresponding to the online changes of learning in the 25 blocks. We averaged the 25 online learning scores to obtain a single online learning score for each participant. To calculate the offline change of statistical learning, we subtracted the learning score of the last bin from the first bin of the next block (the change from the end of the block and the beginning of the next block). Twenty-four scores were obtained corresponding to the offline changes of learning in the 25 blocks (henceforth referred to as “change scores”). We averaged over the 24 offline learning scores to obtain a single offline learning score for each participant. The same procedure was repeated to obtain the online and offline changes for general skill learning, except that scores were obtained from median RTs independent of the probability of items.

### Statistical analysis

Statistical analysis was performed in JASP 0.16. Before conducting the statistical analyses of the main hypotheses, we calculated the mean and median rest duration of the self-paced group. The mean rest period in the self-paced group was 16.67 s (SD* *=* *25.48), and the median rest period was 10.58 s. One-sample *t* tests revealed that the mean rest duration of the self-paced group did not differ significantly from the rest duration of the 15 s group (*t*_(87)_ = 0.62, *p *=* *0.54) but significantly differed from the rest duration of the 30 s group (*t*_(87)_ = −4.91, *p *<* *0.001).

The learning blocks of the ASRT task were grouped into five larger units of analysis (blocks 1–5, blocks 6–10, blocks 11–15, blocks 16–20, and blocks 21–25). Mixed-design ANOVAs on median RTs and statistical learning scores were performed to compare general skill learning and implicit statistical learning between groups, respectively. Offline and online changes were also compared with mixed-design ANOVAs separately for statistical learning and general skill learning. To evaluate the PDP task, we used one-sample *t* tests to compare the proportion of high-probability triplets in the inclusion and exclusion condition to the chance level and conducted mixed-design ANOVA to compare the proportions between groups and conditions. Greenhouse–Geisser corrections were applied if necessary. For ANOVAs, significant main effects and interactions were further analyzed using Bonferroni-corrected *post hoc* comparisons and/or one-sample *t* tests.

In addition to the classical frequentist approach, Bayesian ANOVAs were also performed with the same factors as described above. Here, we report the exclusion Bayes factors (BFs) of Bayesian model averaging across all matched models. BF_exclusion_ indicates the amount of evidence for the exclusion of a given factor. Accordingly, the higher the BF_exclusion_ value (>1), the more it supports the exclusion of the given factor, and, vice versa, the smaller the BF_exclusion_ value (<1), the more evidence for inclusion.

## Results

### Did rest period duration influence statistical learning?

**Figure 2.**
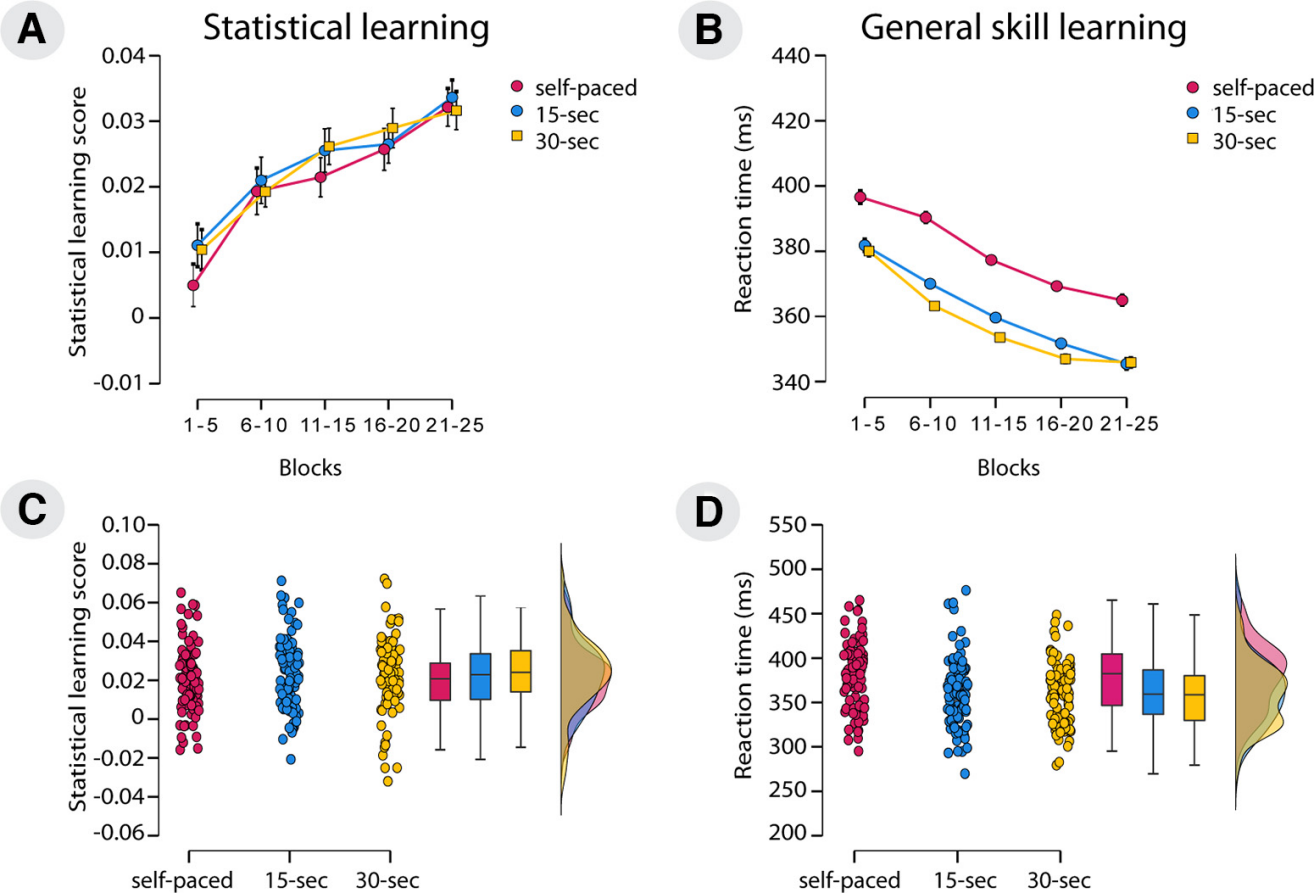
The effect of manipulating rest period duration on statistical learning and general skill learning. Error bars represent the SEM. The *x*-axes indicate the blocks of experiment/experimental groups; the *y*-axes represent the statistical learning score/reaction time. ***A***, The temporal dynamics of statistical learning scores in the three groups. All groups showed a significant increase in statistical learning throughout the experiment, but the learning of the three groups did not differ. To see the original variables that statistical learning score were calculated from, see Extended Data Figure 2-3. ***B***, The temporal dynamics of general skill learning in the three groups. All groups showed a decrease in RT over the course of the experiment, suggesting the learning of general skills. The self-paced group showed slower RTs compared with the 15 and 30 s groups. ***C***, Individual data of the overall statistical learning scores (one dot represents the mean statistical learning score for one participant). Boxplots and violin plots visualize the distribution of statistical learning scores in the three groups. ***D***, Individual data of the general RT scores (one dot represents the mean general RT for one participant). Boxplots and violin plots visualize the distribution of general RTs in the three groups. These results stayed intact without age-based exclusion (Extended Data Figs. 2-1, 2-2).

To test whether the duration of rest periods between learning blocks affected statistical learning, we conducted a mixed-design ANOVA with the within-subjects factor of Blocks (blocks 1–5 vs blocks 6–10 vs blocks 11–15 vs blocks 16–20 vs blocks 21–25) and the between-subjects factor of Group (self-paced, 15 s breaks, 30 s breaks) on the learning scores. The analyses revealed a gradual increase of learning scores in each group, regardless of the rest period duration (main effect of Blocks: *F*_(4,1060)_ = 25.68, *p *<* *0.001, η*_p_*^2^ = 0.09, BF_exclusion_ < 0.001). According to pairwise comparisons, there was no significant increase in learning between blocks 6–10 and blocks 11–15 (*p *=* *0.82), between blocks 6–10 and blocks 16–20 (*p *=* *0.06), between blocks 11–15 and blocks 16–20 (*p *<* *0.99), and between blocks 16–20 and blocks 21–25 (*p *=* *0.19). All other paired comparisons of block arrays were significant (all *p *<* *0.01). Thus, the consecutive learning units did not significantly differ from each other but learning could be discovered between temporally more distant parts of the task. Importantly, the three experimental groups did not differ in statistical learning (main effect of Group: *F*_(2,265)_ = 0.65, *p *=* *0.53, η*_p_*^2^ < 0.01, BF_exclusion_ = 31.39). The Blocks × Group interaction was also nonsignificant (*F*_(8,1060)_ = 0.28, *p *=* *0.97, η*_p_*^2^ < 0.01, BF_exclusion_ = 3 262.88); thus, the three groups did not differ in the time course of statistical learning either (Fig. 2*A*,*C*). To see Results without age-based exclusion, check Extended Data Figure 2-1. Original high-probability and low-probability variables can be seen in Extended Data Figure 2-3.

10.1523/ENEURO.0228-22.2022.f2-1 Figure 2-1 The results of statistical learning without age-based exclusion. We have excluded 11 participants from the main analyses to equalize the mean age between groups to ensure that age-related differences have no effect on our results. To test whether the results of statistical learning are biased by exclusions, we run the same ANOVA without exclusions. The results shown in Figure 2 stayed intact. Download Figure 2-1, DOCX file.

10.1523/ENEURO.0228-22.2022.f2-2 Figure 2-2 The results of general skill learning without age-based exclusion. We have excluded 11 participants from the main analyses to equalize the mean age between groups to ensure that age-related differences have no effect on our results. To test whether the results of general skill learning are biased by exclusions, we run the same ANOVA without exclusions. The results shown in Figure 2 stayed intact. Download Figure 2-2, DOCX file.

**Figure 3.**
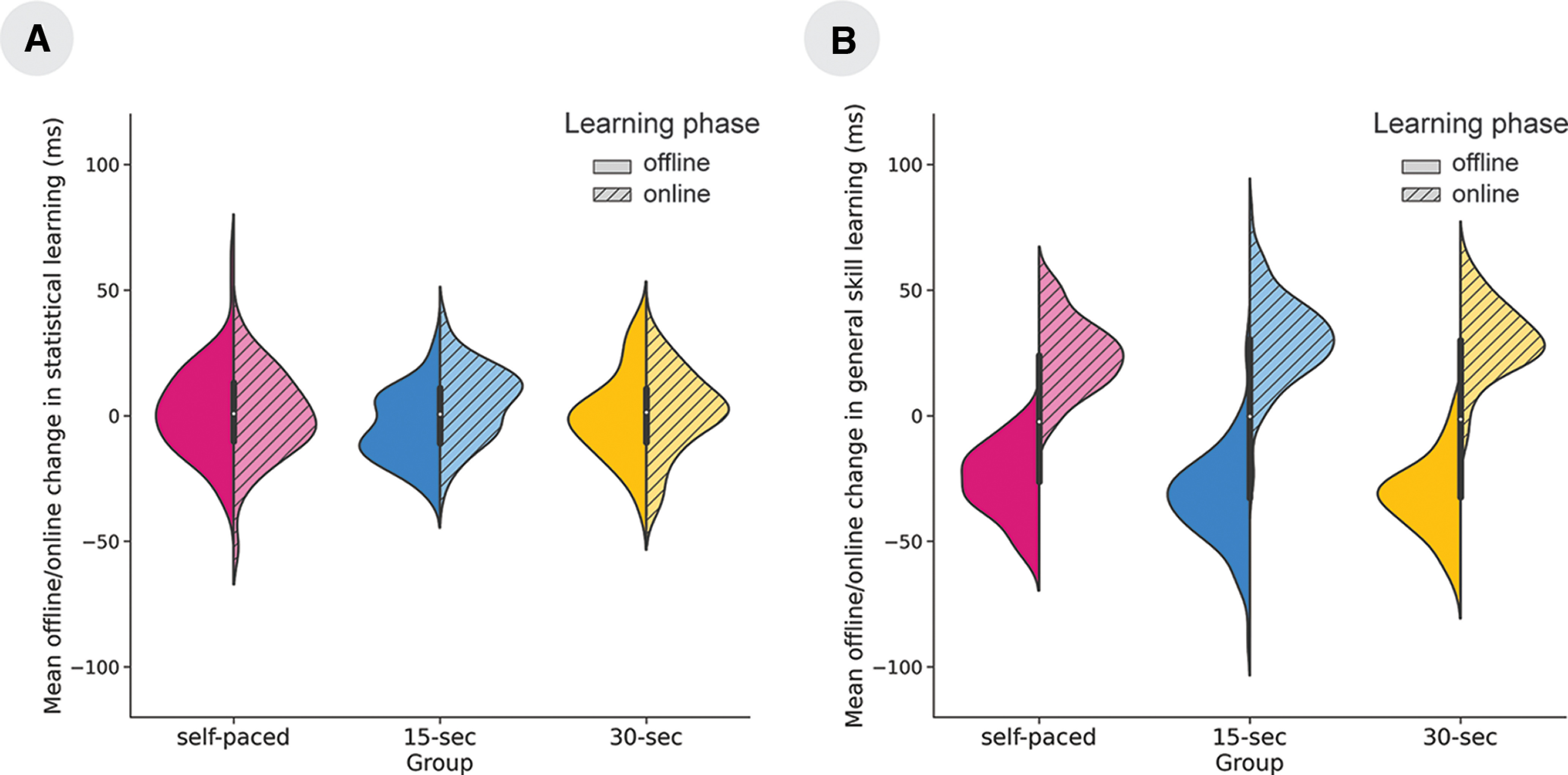
The offline versus online changes in statistical learning/general skill. The *x*-axes indicate the three groups, and the *y*-axes represent the mean offline/online changes in milliseconds. The filled halves of the violin plots indicate offline changes, whereas the striped halves show the online changes. ***A***, In the 15 s group, the offline and online changes differed from each other in statistical learning: the online changes were significantly higher than the offline changes. The group-level online changes were positive (indicating online improvement in statistical learning), whereas the offline changes were negative (indicating forgetting; for dynamic change of the original variables, see Extended Data Fig. 3-3). ***B***, Changes in general skills were similar in the three groups: acceleration after the offline periods and deceleration during the online period. These results stayed intact without age-based exclusion (Extended Data Figs. 3-1, 3-2).

10.1523/ENEURO.0228-22.2022.f2-3 Figure 2-3 Performance of high-probability and low-probability triplets in the three groups. Figure 2 shows the calculated statistical learning scores, while here we depicted the original high-probability (empty circles) and low-probability (filled circles) triplet variables in each group. The *y*-axes indicate the median RT. The *x*-axes show the blocks grouped by five. The error bars represent the 95% confidence interval. Download Figure 2-3, TIF file.

### Did rest period duration influence the performance of general skill learning?

To test whether the overall speedup on the task differed between groups (i.e., whether the duration of rest periods between learning blocks affected general skill learning), we conducted a mixed-design ANOVA with the within-subjects factor of Blocks (blocks 1–5 vs blocks 6–10 vs blocks 11–15 vs blocks 16–20 vs blocks 21–25) and the between-subjects factor of Group (self-paced, 15 s breaks, 30 s breaks) with median RT as the dependent variable. We found a gradual decrease in RTs throughout the task (main effect of Blocks: *F*_(2.73,723.72)_ = 275.21, *p *<* *0.001, η*_p_*^2^ = 0.51, BF_exclusion_ < 0.001). Based on pairwise comparisons, each epoch significantly differed from each other (all *p *<* *0.01), with increasing learning through all blocks. The three groups significantly differed in response times (main effect of Group: *F*_(2,265)_ = 8.69, *p *<* *0.001, η*_p_*^2^ = 0.06, BF_exclusion_ = 0.01), with the self-paced group being slower than the 15 and 30 s groups. The Blocks × Group interaction was also significant (*F*_(8,1060)_ = 2.33, *p *=* *0.04, η*_p_*^2^ = 0.02, BF_exclusion_ = 5.25). Pairwise comparisons revealed significantly higher RTs in the self-paced compared with the 30 s group in blocks 6–10, blocks 11–15, blocks 16–20, and blocks 21–25 (all *p *<* *0.01). The self-paced group also showed significantly higher RTs compared with the 15 s group in blocks 6–10, blocks 11–15, blocks 16–20, and blocks 21–25 (all *p *<* *0.01). Thus, the three groups showed a similar speed in the first learning unit, but the self-paced group began to slow down compared with the other two groups starting from the second learning unit (Fig. 2*B*,*D*). However, the BF_exclusion_ score of the interaction is >3, which indicates moderate evidence for the lack of interaction; thus, the interaction is deemed unreliable. For results without age-based exclusion, see Extended Data Figure 2-2.

### How did break duration affect offline and online statistical learning?

A mixed-design ANOVA was run with the within-subjects factor of Learning Phase (offline vs online) and the between-subject factor of Group (self-paced, 15 s breaks, and 30 s breaks) on the change scores of statistical learning. The ANOVA revealed an interaction between Learning Phase and Group factors (*F*_(2,265)_ = 3.51, *p *=* *0.03, η*_p_*^2^ = 0.03, BF_exclusion_ = 0.05). Bonferroni-corrected *post hoc* comparisons revealed that online and offline changes differed in the 15 s break group (*p *=* *0.04): the offline changes were significantly smaller than the online changes. No main effect of Group (*F*_(2,265)_ = 1.60, *p *=* *0.20, η*_p_*^2^ = 0.01, BF_exclusion_ = 42.28] or Learning Phase (*F*_(2,265)_ = 2.50, *p *=* *0.12, η*_p_*^2^ < 0.001, BF_exclusion_ = 0.97) was found (Fig. 3). For results without aged-based exclusion, check Extended Data Figure 3-1. To see how offline and online learning scores dynamically change across blocks, check Extended Data Figure 3-3.

10.1523/ENEURO.0228-22.2022.f3-1 Figure 3-1 The results of offline versus online statistical learning without age-based exclusion. We have excluded 11 participants from the main analyses to equalize the mean age between groups to ensure that age-related differences have no effect on our results. To test whether the results of offline-online statistical learning are biased by these exclusions, we run the same ANOVA without exclusions. The results shown in Figure 3 stayed intact. Download Figure 3-1, DOCX file.

10.1523/ENEURO.0228-22.2022.f3-3 Figure 3-3 Dynamic change of offline and online learning scores across all blocks in each group. The *y*-axis indicates the mean learning score. The *x*-axis shows the blocks. The error bars represent the 95% confidence interval. There are only 24 blocks because offline learning score could not be calculated for the first block. It could be seen that the 15 s group is the only one where online learning scores are consistently higher than offline learning scores throughout the task. Download Figure 3-3, TIF file.

10.1523/ENEURO.0228-22.2022.f3-2 Figure 3-2 The results of offline versus online general skill learning without age-based exclusion. We have excluded 11 participants from the main analyses to equalize the mean age between groups to ensure that age-related differences have no effect on our results. To test whether the results of offline-online general skill learning are biased by these exclusions, we run the same ANOVA without exclusions. The results shown in Figure 3 stayed intact. Download Figure 3-2, DOCX file.

**Figure 4.**
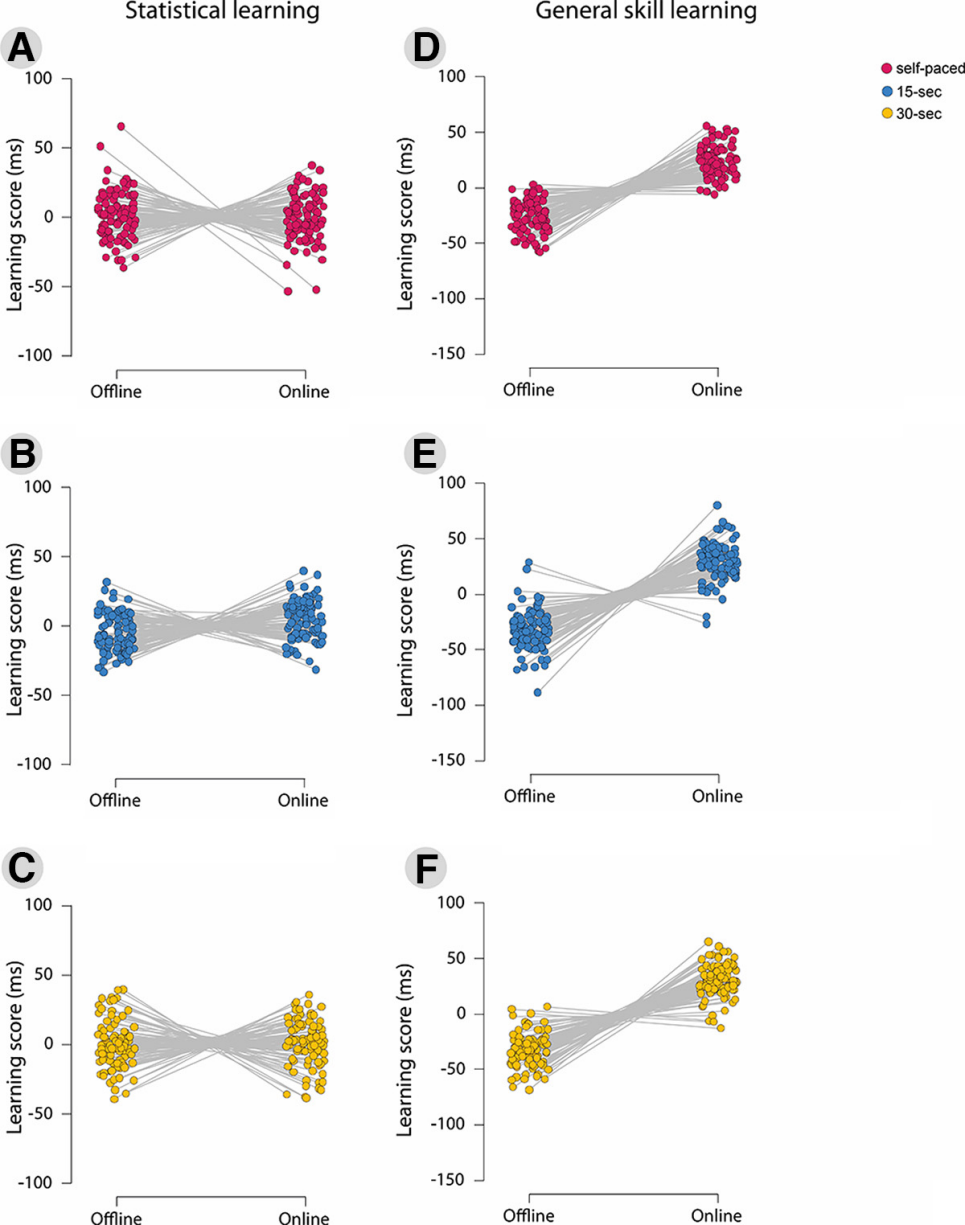
Dynamics of offline/online statistical learning/general skills and forgetting in the different groups. The *y*-axes indicate offline and online learning in milliseconds; the *x*-axes show the mean offline/online learning score of each participant. ***A–F***, The different figures depict the individual data of offline and online statistical learning scores in the self-paced group (***A***), the 15 s group (***B***), and the 30 s group (***C***), and the individual data of offline and online general skill learning scores in the self-paced group (***D***), the 15 s group (***E***), and the 30 s group (***F***). The exact distribution of positive and negative offline and online learning scores can be seen in Extended Data Figures 4-1 and 4-2.

To clarify whether offline and online learning occurred in the whole sample as well as in the three groups, one-sample *t* tests were conducted. We have found that on the whole sample, the online learning scores were significantly different from zero (*t*_(267)_ = 2.05, *p *<* *0.05), while the offline learning score was not (*t*_(267)_ = 1.11, *p *=* *0.27). In the self-paced group, neither the online learning score (*t*_(87)_ = −0.17, *p* = 0.86) nor the offline learning scores (*t*_(87)_ = 0.92, *p *=* *0.36) differed from zero. Similarly, in the 30 s group, neither the online learning scores (*t*_(89)_ = 0.61, *p *=* *0.55) nor the offline learning scores (*t*_(89)_ = −0.05, *p *=* *0.96) differed from zero. However, both learning scores differed from zero in the 15 s group: the online learning scores were higher than zero (*t*_(89)_ = 3.50, *p *<* *0.001), while the offline learning scores were below zero (*t*_(89)_ = −3.39, *p *<* *0.01). We can conclude that in this group, participants learned online and forgot offline. We suggest that the reason behind the lack of online and offline learning in the other two groups can be explained by the balanced ratio of positive and negative learning scores within the groups (Fig. 4*A–C*). The distribution of high positive (≥5) and high negative (≤5) offline and online learning scores can be seen in Extended Data Figures 4-1 and 4-2.

10.1523/ENEURO.0228-22.2022.f4-1 Figure 4-1 The distribution of positive and negative offline statistical learning scores in the groups. As offline and online statistical learning scores in the self-paced and 30 s groups were not different from zero at a group level, we have checked whether there is a difference within the groups between the numbers of online and offline learning and forgetting scores. We compared the distribution of those who had high positive (≥5) or high negative (less than or equal to –5) offline learning scores in the three groups. The distribution of those who learned or forgot offline is in balance in the self-paced and the 30 s groups, which could result in no offline learning at the group level. However, in the 15 s group, more participants forgot than learned offline, which resulted in offline forgetting at a group level. Download Figure 4-1, DOCX file.

10.1523/ENEURO.0228-22.2022.f4-2 Figure 4-2 The distribution of positive and negative online learning scores in the groups. Distributions of high positive (≥5) and high negative (less than or equal to –5) learning scores in the three groups were tested for online learning. Similar to offline learning, in the self-paced and the 30 s group, the proportions of those who learned or forgot online are similar, while in the 15 s group, almost twice as many participants learned as forgot online. Download Figure 4-2, DOCX file.

### How did break duration affect offline and online general skill learning?

A mixed-design ANOVA was run on the change scores of general skill learning, with the within-subjects factor of Learning Phase (offline vs online) and the between-subject factor of Group (self-paced, 15 s breaks, 30 s breaks). We found the main effect of Learning Phase (*F*_(2,265)_ = 920.49, *p *<* *0.001, η*_p_*^2^ = 0.77, BF_exclusion_ < 0.001), with a slowing down of RT during the blocks, while an acceleration of RTs occurred after the rests. No main effect of Group was found (*F*_(2,265)_ = 0.02, *p *=* *0.98, η*_p_*^2^ < 0.001, BF_exclusion_ = 45.61). The interaction between the Learning Phase and Group factors was significant (*F*_(2,265)_ = 4.38, *p *=* *0.01, η*_p_*^2^ < 0.03, BF_exclusion_ = 0.01). However, no differences survived Bonferroni-corrected between-group comparisons for online and offline changes (all comparisons between groups revealed *p *>* *0.17; Fig. 3). For results without age-based exclusion, see Extended Data Figure 3-2.

One-sample *t* tests revealed that participants learned online (*t*_(267)_ = 29.14, *p *<* *0.001) and forgot offline (*t*_(267)_ = −30.60, *p *<* *0.001) the general skill in the whole sample. This pattern was observed in all three groups. Online learning of the general skill took place in the self-paced group (*t*_(87)_ = 15.87, *p *<* *0.001), in the 15 s group (*t*_(89)_ = 16.35, *p *<* *0.001), as well as in the 30 s group (*t*_(89)_ = 19.18, *p *<* *0.001). During the offline periods, participants’ general skill performance decreased in the self-paced group (*t*_(87)_ = −17.16, *p *<* *0.001), the 15 s group (*t*_(89)_ = −16.78, *p *<* *0.001, and the 30 s group (*t*_(89)_ = −20.21, *p *<* *0.001; Fig. 4*D–F*).

### Was statistical learning implicit in the three groups?

Last, we tested whether learning occurred implicitly in the three experimental groups. We compared the percentage of high-probability triplets generated in the PDP task to the chance level (25%) in the three groups. Participants in the self-paced group generated more high-probability triplets both in the inclusion and exclusion conditions than would occur at chance level (inclusion condition: mean ± SD = 31.5 ± 0.8%, *t*_(80)_ = 7.79, *p *<* *0.001, BF_01_ = 0.001; exclusion condition: mean ± SD = 29.2 ± 1%, *t*_(80)_ = 3.06, *p* < 0.001, BF_01_ < 0.001). It was the same in the 15 s group (inclusion condition: mean ± SD = 31.1 ± 0.8%, *t*_(86)_ = 7.54, *p *=* *0.001, BF_01_ < 0.001; exclusion condition: mean ± SD = 27.5 ± 1%, *t*_(86)_ = 2.62, *p *=* *0.001, BF_01_ < 0.001), and in the 30 s group (inclusion condition: mean ± SD = 30.3 ± 0.7%, *t*_(86)_ = 6.57, *p *<* *0.001, BF_01_ < 0.001; exclusion condition: mean ± SD = 29.0 ± 1%, *t*_(86)_ = 3.30, *p *=* *0.001, BF_01_ < 0.001). Thus, we can conclude that learning can be considered implicit in all groups.

Furthermore, we explored the potential differences between groups with a 2 (condition: inclusion vs exclusion) × 3 (group: self-paced vs 15 s vs 30 s) ANOVA. The main effect of the condition was significant (*F*_(1,252)_ = 15.027, *p *=* *0.001, η*_p_*^2^ = 0.06, BF_exclusion_ = 0.01), indicating that participants performed better in the inclusion condition. The group main effect did not reach significance (*F*_(2,252)_ = 0.13, *p *=* *0.88, η*_p_^2^* = 0.001, BF_exclusion_ = 28.02), indicating that the three groups performed equally on the tasks. The interaction of the condition and group factors was also nonsignificant *F*_(2,252)_ = 1.03, *p *=* *0.36, *η_p_*^2^ = 0.01, BF_exclusion_ = 9.35), revealing that the lack of difference between groups was not influenced by the task condition. Together, the results indicate that the knowledge of the three groups remained equally implicit.

## Discussion

Our study aimed at testing whether the duration of short rest periods, when neural replay occurs, influences statistical learning and general skill learning. To measure these two aspects of learning independently, we used an implicit sequence-learning task, the ASRT task (Howard et al., 2004). We varied the lengths of rest periods across participants: 15 s (15 s group) or 30 s (30 s group) between the learning blocks or participants could decide when to resume the task (self-paced group). We wondered (1) whether the three groups differed in the extent of general skill learning and statistical learning and (2) whether rapid consolidation emerged during between-block rest periods in general skill learning and statistical learning. Break duration affected general skills and statistical learning differently. We observed that the self-paced group was generally slower than the other two groups. However, all groups showed a similar degree of statistical learning. Because of the same proportion of those who learned or forgot offline/online, group-level offline and online learning could not be detected in the self-paced and 30 s groups, while the 15 s group showed mainly online improvement and offline forgetting.

Our results suggest that the duration of rest periods is not necessarily decisive in statistical learning over the entire task. This result seems to be inconsistent with the results of the study by Bönstrup et al. (2019). They showed that short, 10 s rest periods could facilitate motor skill learning, and this improvement could continue with even shorter rest periods (Bönstrup et al., 2020). In contrast, previous studies that also measured pure statistical learning are consistent with our results (Fanuel et al., 2022). The task used in the study of Bönstrup et al. (2020) does not allow the differentiation of subprocesses of learning and mixes general skill learning with statistical learning; therefore, it is difficult to decide which was the determining factor in this result.

According to the results of Buch et al. (2021), we expected that the longer rest period (i.e., 30 s) would result in better learning compared with the shorter rest period (i.e., 15 s), because it may contain more replays, which is the neural basis of rapid consolidation. However, we measured only one learning session, and it is conceivable that the beneficial effect of an expanded rest period would appear instead in the longer run. On the other hand, the length of rest periods used in our study might not have been suitable to capture the critical period when rapid consolidation is beneficial in statistical learning. These questions should be further explored using a much more comprehensive range of rest periods and introducing delayed testing of implicit statistical learning.

In general skill learning, participants showed longer RTs in the self-paced condition where they were allowed to decide about the rest period duration, compared with those conditions where rest period duration was fixed (i.e., 15 and 30 s). How could we interpret the longer RTs in the self-paced group? On the one hand, this difference could be because of a difference in the rest period duration in the self-paced group compared with the two fixed rest period groups. On the other hand, it could be because of the specificity of the self-paced condition. The mean rest period in the self-paced group was similar to the rest period duration of the 15 s group, but the two groups still significantly differed in overall speed. However, the high SD shows considerable variability in the time the participants decided to rest between blocks. We suggest that it is not the duration of the rest period that is critical in the performance of general skill learning, but the nature of the expiry of the rest period (voluntary or compulsory). The knowledge that the rest period will be limited might have urged participants to complete the task as soon as possible, which resulted in faster RTs.

Our results about the offline and online changes in general skill learning are in accordance with previous studies with the same sequence-learning task (Quentin et al., 2021; Fanuel et al., 2022): during practice, the speed decreases, and between blocks, it increases. However, our results only partially replicated previous findings on statistical learning. Previously, it was shown that statistical learning mainly occurs during blocks, and forgetting occurs between blocks. In our study, this pattern was only detectable for the 15 s group: in the 30 s and self-paced groups, such strong dissociation could not be seen in the online versus offline changes. It is possible that the 30 s and the self-paced groups took enough break time to benefit from both online learning and rapid consolidation (potentially allowing more replay to occur in the offline periods), but the fixed 15 s length was not enough for the latter. This hypothesis could be supported by the results of the study by Prashad et al. (2021), who found offline improvement in probabilistic sequence learning with 2-min-long breaks between the learning blocks. However, as the differences in break durations are relatively large between these two studies, it is still a pending question to establish the minimum length of a between-block break for rapid consolidation. Studies that directly manipulate the number of neural replays between block periods are warranted.

Another possible explanation might be that our study was completed online: participants completed the task in their environment, where the stress level is possibly smaller than in laboratory settings. The limited rest period could have increased the stress level during the experiments, creating similar circumstances that participants experienced in the laboratory. As statistical learning is affected by stress levels (Tóth-Fáber et al., 2021), this could have prompted participants to maximize their performance during practice and benefit more from rest periods. However, no difference in learning outcomes was found between the groups, suggesting that different lengths of the rest periods only change the learning dynamics; they do not affect the outcomes of statistical learning.

Together, we observed that the manipulation of the length of the rest periods—indirectly the neural replay—affects general speed on a sequence-learning task. In contrast, statistical learning seems to be independent of the length of the rest period. The length of rest periods did not affect the outcome of statistical learning, but did affect the dynamics of learning (i.e., whether learning occurs online or offline): if we do not have enough time during breaks for offline consolidation, we might compensate by increasing online learning performance. Thus, our results suggest that the length of short rest periods has a different effect on separate learning and consolidation processes. Also, from a methodological perspective, our results show the importance of measuring the temporal dynamics of learning, and do not provide only a general measure of the overall learning across the task.

## Synthesis

Reviewing Editor: Christina Zelano, Northwestern University - Chicago Decisions are customarily a result of the Reviewing Editor and the peer reviewers coming together and discussing their recommendations until a consensus is reached. When revisions are invited, a fact-based synthesis statement explaining their decision and outlining what is needed to prepare a revision will be listed below. The following reviewer(s) agreed to reveal their identity: Mircea Van Der Plas.

The reviewers agreed that this study addresses an important topic, and that the findings will advance the field and our understanding of motor and cognitive skills. Both reviewers raised some important concerns about the methods and statistical analyses. These concerns must be addressed in full before the manuscript can be considered for publication. Additionally, some clarifications in the terminology used throughout the manuscript will be helpful. See detailed comments below:

Reviewer 1:

In this study, the authors examined whether the rest period length affects rapid online and offline statistical learning and general motor improvement in the ASRT task. It was found that the length of rest did not affect statistical learning, while it results in different formats (online vs. offline) of learning such statistical structures. This study tackled an important question in cognitive and motor learning, the experiment was well designed and the paper was clearly written.

Several issues are listed below, raising concerns about the analysis methods. In addition, there was insufficient consideration of the published literature on rapid online and offline learning of probabilistic sequences.

There are several concerns regarding the analyses. Learning blocks were combined into groups of 5 blocks when analyzing statistical learning and general motor improvement. However, measurements were averaged across all 25 blocks when analyzing offline and online learning. Why the authors chose this specific way to analyze online and offline learning is unclear. To better understand offline and online learning, it would be helpful to see how offline and online learning dynamically change across blocks. This is especially important for the self-paced rest group because the magnitude of online and offline learning may depend on the exact length of rest participants chose after each block. Since there were about 300 participants, this study should have enough power for this analysis.

Overall RT of all trials was used to reflect general motor improvement. However, these trials included the high probability triplets and their RTs were primarily driven by statistical learning. Thus, the way how the authors assessed general motor improvement was likely to be heavily contaminated by the effect of statistical learning. RT of the lower probability triplets would be a better indicator of general motor improvement.

Relatedly, statistical learning was quantified by the RT difference between high and low probability triplets. It would be helpful to see the performance of these original variables (RT of high or low probability triplets). Is there any RT improvement in the low probability triplets?

Some trials were excluded from analyses (line 178). Statistics of these trials should be reported. How many trials were excluded? How many error trials?

The authors concluded that the self-paced and 30-sec group benefited from online and offline learning equally for statistical learning.” The results (line 276 - 284) did not support this conclusion. Were the online and offline magnitudes different from zero? Again, the authors need to report the details of the original variables instead of only reporting some values derived from these variables.

More careful consideration of the published literature is needed. The paper is majorly grounded on Bonstrup 2020 paper using an explicit learning task and two papers on implicit learning using the ASRT task (Fanuel et la., 2022 & Quentin et al., 2021). The latter two papers suggest that implicit learning occurs online and does not benefit from offline learning. A recent paper missing here is very related to rapid memory consolidation of implicit statistical learning (Prashad et al. 2022; Sequence Structure Has a Differential Effect on Underlying Motor Learning Processes), which reported offline learning of probabilistic sequences and such offline learning was not because of general motor improvement. The results are inconsistent with the finding of this current paper. A discussion regarding these results will be helpful.

Memory consolidation, memory stabilization, and offline learning seem to be used interchangeably in this paper. However, they are not the same. More accurate use of these terms would be helpful to the reader.

Reviewer 2:

In this study the authors have attempted to replicate and further investigate the phenomenon, that consolidation can occur on very small time-scales, where even short breaks may lead to performance improvements. Hypothetically, if rapid consolidation occurs on would expect a longer break to lead to more consolidation and therefor more enhancement in the offline period. In this manuscript there is no suggestion that rapid consolidation depends on time, based on a shorter window 0f 15 s and a longer window of 30 s as well as a self-paced break which tends to be on the shorter end of he other two windows. This finding quite trivial if one considers that there seems to be no real evidence of rapid consolidation in the first place. At least based on the presented data, there seems to be no systematic benefit of the breaks in general, if a difference is observed, a break seems to actually be detrimental to learning performance, both in terms of statistical learning as well as general performance. However, the authors do not make this as explicit in the discussion, as evidenced by phrases such as 'The self-paced group and 30-sec group benefitted from offline and online improvements in statistical learning equally'. When one takes a look at the figures , however, this seems to be due to the fact that the distributions (as presented in figure 3) do not significantly differ from 0. It would be quite important to verify that whether there is any offline learning at all before continuing with the rest of the analysis as this is the core assumption of this manuscript. This would also have many implications of how the discussion is phrased.

There is also a few methodological issues I would like to bring up. The authors have decided to run a repeated measures ANOVA to analyse the progression of both statistical and general skill learning throughout the experiment. For this they block the data into blocks of 5 blocks. Why was this procedure done? Instead they could have used all blocks and even made the analysis explicitly a regression (which is mathematically equivalent to the ANOVA, but give a finer description of the data if one does not block it the way it has been done in the current version). If would be interesting to see a justification from the authors for this specific approach they have taken to analyse the data.

Moreover, the authors have decided to include a Bayesian analysis. However, the results of that analysis are not discussed. For example, in the General skill learning ANOVA a Block x Group interaction was observed in the frequentist analysis with a p-value of 0.04, however, the Bayes factor is at odds with this conclusion and actually suggests the opposite with a BF>1. This would actually cast doubt on the validity of this interaction effect. This part of the analysis is not discussed anywhere, making the BF factor a bit of an afterthought. If they would like to keep this aspect of the analysis, I would suggest that they actually discuss the implications of this addition or leave it out completely.

Lastly, the authors decide to subtract the median reaction time as part of their normalization procedure and dividing by the mean, instead of the more traditional methods of subtracting by the mean. Is there a particular reason for this choice?

## Author Response

Rewiever #1

1) “Learning blocks were combined into groups of 5 blocks when analyzing statistical learning and general motor improvement. However, measurements were averaged across all 25 blocks when analyzing offline and online learning. Why the authors chose this specific way to analyze online and offline learning is unclear. To better understand offline and online learning, it would be helpful to see how offline and online learning dynamically change across blocks. This is especially important for the self-paced rest group because the magnitude of online and offline learning may depend on the exact length of rest participants chose after each block. Since there were about 300 participants, this study should have enough power for this analysis.”

Thank you for this suggestion. The reason behind our decision to calculate one offline /online learning score for each participant is that we would not have had enough statistical power for a block-by-block analysis. Although 268 participants seem to provide enough power, in such ANOVA, there would be 3 (group) × 2 (offline vs. online) × 24 (blocks) factors, resulting in just one or two participants per factor. However, an ANOVA like the one used to analyse the ASRT learning indicators could be misleading, since the dynamic changes in offline/online scores are not as consistent as for the overall learning progress. Thus, treating five consecutive blocks together may not be logical. Furthermore, the analysis plan was pre-registered, and we did not want to deviate from it.

Nevertheless, we agree that it could be important information how offline and online learning dynamically change across all blocks in the separate groups. Hence, we conducted three ANOVAs with the offline and online learning scores of all blocks separately for each group.

In the self-paced group, there was not significant neither the main effect of Blocks (F(10.56, 401.26) = 0.85, p = .59, ηp2 = .02), nor the main effect of Learning Phase (F(1, 38) = 0.88, p = .35, ηp2 = .02). The Blocks × Learning Phase interaction was also not significant (F(12.82, 487.30) = 0.69, p = .77, ηp2 = .02).

In the 15-sec group, the main effect of Blocks was also not significant (F(9.98, 468.87) = 0.46, p = .92, ηp2 = .01). The main effect of Learning Phase was significant only in this group (F(1, 47) = 7.57, p < .01, ηp2 = .14). However, the Blocks × Learning Phase interaction was not significant F(13.71, 644.47) = 0.97, p = .48, ηp2 = .02).

In the 30-sec group, there was not significant neither the main effect of Blocks (F(10.82, 486.66) = 0.77, p = .67, ηp2 = .02), nor the main effect of Learning Phase (F(1, 45) = 0.01, p = .94, ηp2 < .001). The Blocks × Learning Phase interaction was also not significant (F(13.51, 607.93) = 1.02, p = .43, ηp2 = .02).

The results of the three ANOVAs confirm no effect of time on offline and online learning. We also depict the dynamic change of offline and online learning across all blocks in the different groups (see Figure 1).

Figure 1. Dynamic change of offline and online learning scores across the 24 blocks in each group. The y-axes indicates the mean learning score. The x-axes shows the blocks. The error bars represent 95% confidence interval.

We can see in Figure 1 that the 15-sec group is the only one where online learning scores are consistently higher than offline learning scores throughout the task, which is also supported by the results of the ANOVA.

We included the results of the three ANOVAs and this figure as Extended data (see Figure 3-3, Figure 3-4).

Figure 3-4 The results of ANOVAs for offline and online changes across all blocks in the separate groups. To see how offline and online statistical learning dynamically change across all blocks in the separate groups, we conducted three ANOVAs with the offline and online statistical learning scores of all blocks separately for each group. The results of the three ANOVAs confirm no effect of time on offline and online learning, as the main effect of Blocks was not significant in either group. Download Figure 3-4, DOCX file.

2) “Overall RT of all trials was used to reflect general motor improvement. However, these trials included the high probability triplets and their RTs were primarily driven by statistical learning. Thus, the way how the authors assessed general motor improvement was likely to be heavily contaminated by the effect of statistical learning. RT of the lower probability triplets would be a better indicator of general motor improvement.”

Thank you for this comment. Indeed, we operationalized statistical learning by comparing performance on high- and low-probability triplets and general skill learning as overall reaction time independently of triplet types, according to previous ASRT studies (Fanuel et al., 2022; Hallgató et al., 2013; Hedenius et al., 2013; Kobor et al., 2017; Nemeth et al., 2010; Nemeth and Janacsek, 2011; Quentin et al., 2021; Twilhaar et al., 2019). However, we have checked if our results are similar when measuring general skill learning using only low-probability trials and our results are the same. The main effect of Block remained significant (F(3.09, 819.61) = 150.59, p < .001, ηp2 = .36, BFexclusion < 0.001), as is the main effect of Group (F(2, 265) = 8.57, p < .001, ηp2 = .06, BFexclusion = 0.01). The interaction also remained significant (F(8, 1060) = 2.08, p = .04, ηp2 = .02, BFexclusion = 2.80) with a lower BF value than it is in the original analysis (BFexclusion = 5.25), but still favors the exclusion of interaction from the model.

Figure 2. General skill learning with the measure of RTs on low-probability triplets. The y-axis indicates the median RT of low-probability triplets. The x-axis shows the blocks grouped by five. The error bars represent 95% confidence interval.

Since the results of general skill learning remained the same when measured with only RTs of low-probability triplets, we kept the original analysis in the manuscript in order not to deviate from the pre-registration.

3) “Relatedly, statistical learning was quantified by the RT difference between high and low probability triplets. It would be helpful to see the performance of these original variables (RT of high or low probability triplets). Is there any RT improvement in the low probability triplets?”

Thank you for this comment. Here, we have created a figure where the original low- and high-probability triplet variables are comparable separately in the three groups:

Figure 3. Performance of high-probability (empty circles) and low-probability (filled circles) triplets in the three groups. The y-axes indicate the median RT. The x-axes show the blocks grouped by five. The error bars represent 95% confidence interval.

As shown in the figure, the performance of high- and low-probability triplets is similar in the first learning unit. Although low-probability triplets also show RT improvement, the performance of the two triplet types gradually forks, with faster RTs for the high-probability triplets.

We included this figure in the manuscript as Extended data (see Figure 2-3).

4) “Some trials were excluded from analyses (line 178). Statistics of these trials should be reported. How many trials were excluded? How many error trials?”

Thank you for this question. There was a total of 535.994 trials, from which a total of 49.927 (9.31 %) incorrect trials were excluded. Regarding triplets, 48.715 (9.09 %) was trills (e.g., 1-2-1) and 16.302 (3.04 %) was repetitions (e.g., 1-1-1) of all trials. Furthermore, there were 1304 (0.24 %) of the trials with RT above 1000 ms. As there were overlaps between the trials with different exclusion criteria (e.g., from the 48.715 trills 7544 was also incorrect trials), the total amount of excluded trials is not the sum of the numbers of the different types of exluded trials. We excluded a total amount of 108.344 trials (20.22 % of all trials).

Table 1. The amount of excluded trials

Inaccurate responses Trills Repetitions RTs above 1000 ms Total

49.927 (9.12 %) 48.715 (9.09 %) 16.302 (3.04 %) 1304 (0.24 %) 108.344 (20.22 %)

We included these data in the manuscript too (pp. 9):

„There was a total of 535,994 trials, from which a total of 49,927 (9.31%) incorrect trials were excluded. Regarding triplets, 48,715 (9.09%) were trills (e.g., 1-2-1), and 16,302 (3.04%) were repetitions (e.g., 1-1-1). Furthermore, there were 1304 trials (0.24% of all trials) with RT above 1000 ms. As there were overlaps between the trials with different exclusion criteria (e.g., from the 48,715 trills, 7544 were also incorrect trials), the total amount of excluded trials is not the sum of the numbers of the different types of excluded trials. We excluded a total amount of 108,344 trials (20.22% of all trials).”

5) „The authors concluded that the self-paced and 30-sec group benefited from online and offline learning equally for statistical learning.” The results (line 276 - 284) did not support this conclusion. Were the online and offline magnitudes different from zero? Again, the authors need to report the details of the original variables instead of only reporting some values derived from these variables.”

Thank you for this remark. We have conducted one-sample t-tests in order to check whether the mean online and offline scores differ from zero in the whole sample, as well as in each group. We have found that on the whole sample, the online learning score is significantly different from zero (t(267) = 2.05, p < .05), while the offline learning score is not (t(267) = 1.11, p = .27).

In the self-paced group, neither the online learning score (t(87) = -0.17, p = .86, nor the offline learning score (t(87) = 0.92, p = .36) is different from zero. Similarly in the 30-sec group, neither the online learning score (t(89) = 0.61, p = .55), nor the offline learning score (t(89) = -0.05, p = .96) differs from zero.

However, both learning score differs from zero in the 15-sec group: the online learning score is higher than zero (t(89) = 3.50, p < 0.001), the whilst the offline learning score is smaller than zero (t(89) = -3.39, p < 0.01). We can conclude that in this group participants learned online and forgot offline.

But then, what is the explanation for the fact that in the other two groups, neither online nor offline learning was found on the group level? In Table 2, we compared the distribution of those who had high positive ({less than or equal to}5) or high negative ({greater than or equal to}-5) offline learning scores in the three groups.

Table 2. The distribution of positive and negative offline learning scores in the groups

Group

Self-paced 15-sec 30-sec Total

Learn offline 38 25 27 90

Forget offline 31 49 34 114

Total 69 74 61 204

Chi-square test: χ²(2) = 6.57, p < 0.05-

As we can see, the distribution of those who learned or forgot offline is in balance in the self-paced and the 30-sec group, which could be resulted in no measurable offline learning at a group level. However, in the 15-sec group, more participants forgot than learned offline, which resulted in offline forgetting at a group level.

Distributions in high positive and high negative learning scores were also tested for online learning (see Table 3). Similarly to offline learning, in the self-paced and the 30-sec group, the proportion of those who learned or forgot online is similar, while in the 15-sec group, almost twice as many learned as forgot online.

Table 3. The distribution of positive and negative online learning scores in the groups

Group

Self-paced 15-sec 30-sec Total

Learn online 34 50 34 118

Forget online 38 25 26 89

Total 72 75 60 207

Chi-square test: χ²(2) = 5.67, p = 0.06

We have included the findings of t-tests in the manuscript (pp. 14) and the tables as Extended data (see Figure 4-1 and Figure 4-2). According to these results, we rephrased the quoted sentence from the Discussion section of interpreting the results regarding offline and online changes to the following:

„Due to the same proportion of those who learned or forgot offline/online, group-level offline and online learning could not be detected in the self-paced and 30-sec groups, while the 15-sec group showed mainly online improvement and offline forgetting.” (pp. 17)

6) “More careful consideration of the published literature is needed. The paper is majorly grounded on Bonstrup 2020 paper using an explicit learning task and two papers on implicit learning using the ASRT task (Fanuel et la., 2022 & Quentin et al., 2021). The latter two papers suggest that implicit learning occurs online and does not benefit from offline learning. A recent paper missing here is very related to rapid memory consolidation of implicit statistical learning (Prashad et al. 2022; Sequence Structure Has a Differential Effect on Underlying Motor Learning Processes), which reported offline learning of probabilistic sequences and such offline learning was not because of general motor improvement. The results are inconsistent with the finding of this current paper. A discussion regarding these results will be helpful.”

Thank you for drawing our attention to another relevant paper on the topic. We have found that the result of Prashad and colleagues is not quite the opposite to ours, since they only applied one break length, which is significantly longer than ours. As we suggested that our shortest break group did not show offline improvement due to the insufficient length of breaks (indirectly, the few neural replays), their result seems to be consistent with ours, where longer breaks have also led to offline improvement. In the revised version, we have supplemented the discussion of the results with the work of Prashad and colleagues (2021):

„This hypothesis could be supported by the results of Prashad and colleagues (2021), who found offline improvement in probabilistic sequence learning with two-minute-long breaks between the learning blocks. However, as the difference in break durations are relatively large between these two studies, it is still a pending question to establish the minimum length of a between-block-break for rapid consolidation.” (pp. 19)

7) “Memory consolidation, memory stabilization, and offline learning seem to be used interchangeably in this paper. However, they are not the same. More accurate use of these terms would be helpful to the reader.”

Thank you for this helpful suggestion. We have reviewed the manuscript regarding the terminology and revised it throughout the text in order to be more consistent. The following amendments were made:

• We changed „stabilization processes” to „consolidation processes” (pp. 1),

• „rapid offline improvement” to „rapid consolidation” (pp. 1)

• „offline learning or memory consolidation” to „memory consolidation” (pp. 3)

• „rapid offline gains” to „rapid consolidation” (pp. 4, 18)

• „offline learning” to „rapid consolidation” (pp. 18)

Reviewer #2

1) “In this manuscript there is no suggestion that rapid consolidation depends on time, based on a shorter window of 15 s and a longer window of 30 s as well as a self-paced break which tends to be on the shorter end of he other two windows. This finding quite trivial if one considers that there seems to be no real evidence of rapid consolidation in the first place. At least based on the presented data, there seems to be no systematic benefit of the breaks in general, if a difference is observed, a break seems to actually be detrimental to learning performance, both in terms of statistical learning as well as general performance. However, the authors do not make this as explicit in the discussion, as evidenced by phrases such as ‘The self-paced group and 30-sec group benefitted from offline and online improvements in statistical learning equally’. When one takes a look at the figures , however, this seems to be due to the fact that the distributions (as presented in figure 3) do not significantly differ from 0. It would be quite important to verify that whether there is any offline learning at all before continuing with the rest of the analysis as this is the core assumption of this manuscript. This would also have many implications of how the discussion is phrased.”

Thank you for this comment. Following your indications, we have checked whether mean online and offline learning scores are different from zero in the whole sample, as well as in each group. One sample t-tests revelaed that online learning score is significantly higher than zero (t(267) = 2.05, p < .05) in the whole sample, while offline learning score really does not differ significantly from zero (t(267) = 1.11, p = .27).

Regarding the different groups, we found that online and offline learning scores differ from zero only in the 15-sec group. In this group, participants learned online (t(89) = 3.50, p < 0.001) and forgot offline (t(89) = -3.39, p < 0.01). In the self-paced group, we could not find learning or forgetting in either online (t(87) = -0.17, p = .86) or offline learning (t(87) = 0.92, p = .36). Similarly in the 30-sec group, neither the online learning score (t(89) = 0.61, p = .55) nor the offline learning score (t(89) = -0.05, p = .96) was different significantly from zero.

One possible explanation for this phenomenon is the fact that in the self-paced and the 30-sec group half of the participants showed positive offline and online learning scores, while the other half showed negative offline and online learning scores, which led to the two subsets canceling each other out, resulting in a null effect. We believe that this does not significantly affect the meaning of the results: within the self-paced and 30-sec group offline and online ‘learners’ are indeed equally distributed (which results in a null effect at a group level), while in the 15-sec group learning shifts towards online benefit.

We have included the findings of t-tests in the manuscript (pp. 14):

„To clarify whether offline and online learning occurred in the whole sample as well as in the three groups, one sample t-tests were conducted. We have found that on the whole sample, the online learning score was significantly different from zero (t(267) = 2.05, p < .05), while the offline learning score was not (t(267) = 1.11, p = .27). In the self-paced group, neither the online learning score (t(87) = -0.17, p = .86, nor the offline learning scores (t(87) = 0.92, p = .36) differed from zero. Similarly, in the 30-sec group, neither the online learning scores (t(89) = 0.61, p = .55) nor the offline learning score (t(89) = -0.05, p = .96) differed from zero. However, both learning scores differed from zero in the 15-sec group: the online learning scores were higher than zero (t(89) = 3.50, p < .001), whilst the offline learning scores were below zero (t(89) = -3.39, p < .01). We can conclude that in this group, participants learned online and forgot offline. We suggest that the reason behind the lack of online and offline learning at group level in the other two groups can be explained by the balanced ratio of positive and negative learning scores within the groups (see Figure 4A, Figure 4B, and Figure 4C). The distribution of high positive (>5) and high negative (<5) offline and online learning scores can be seen in the extended data Figure 4-1 and Figure 4-2.”

We also included tables of the distribution of positive and negative offline and online learning scores as Extended data (see Figure 4-1 and Figure 4-2). According to these results, we rephrased the quoted sentence from the Discussion section of interpreting the results regarding offline and online changes to the following:

„Due to the same proportion of those who learned or forgot offline/online, group-level offline and online learning could not be detected in the self-paced and 30-sec groups, while the 15-sec group showed mainly online improvement and offline forgetting.” (pp. 17)

2) „The authors have decided to run a repeated measures ANOVA to analyse the progression of both statistical and general skill learning throughout the experiment. For this they block the data into blocks of 5 blocks. Why was this procedure done? Instead they could have used all blocks and even made the analysis explicitly a regression (which is mathematically equivalent to the ANOVA, but give a finer description of the data if one does not block it the way it has been done in the current version). If would be interesting to see a justification from the authors for this specific approach they have taken to analyse the data.”

Thank you for this suggestion. The main reason why we analyze the variables of the ASRT task divided into larger units (Barnes et al., 2008; Bennett et al., 2007; Nemeth et al., 2010) is the fact that learning occurs with a slow graduation, and the block-by-block learning scores are quite noisy; therefore, a stronger effect can be shown through this analysis method. However, at Reviewer 2’s suggestion, we examined what would be the results of linear regressions.

Firstly, we have run a linear regression for general skill learning with median RTs of blocks as the dependent variable and with the block and group dummy variables as independents. The model was significant (F(3, 6696) = 265.26, p < .001), with R2 = 10.6 %.

Table 4. Coefficients of linear regression for general skill learning

Explanatory variable Unstandardized coefficient Standardized coefficient t

P value

B Std. error Beta

Constant 402.114 1.406 286.035 <.001

Block -1.777 0.078 -0.264 -22.875 <.001

15-sec group -17.778 1.375 -0.173 -12.929 <.001

30-sec group -21.168 1.375 -0.206 -15.394 <.001

We also have run a similar linear regression for statistical learning with the standardized learning scores as the dependent variable, and with the block and group dummy variables as independents. This model was also significant (F(3, 6696) = 26.44, p < 0.001), with R2 = 1.2 %.

Table 5. Coefficients of linear regression for statistical learning

Explanatory variable Unstandardized coefficient Standardized coefficient t

P value

B Std. error Beta

Constant 0.008 0.002 3.633 <.001

Block 0.001 < 0.001 0.106 8.687 <.001

15-sec group 0.004 0.002 0.028 1.962 .050

30-sec group 0.002 0.002 0.016 1.120 .263

The results of regressions are similar to the results of ANOVAs: block and group variables are significant explanatory variables for general skill learning (in the ANOVA, there was a significant group main effect with slower reaction times in the self-paced group), while only the block is a significant explanatory variable for statistical learning (in the ANOVA the groups did not differ in statistical learning).

As the main results stayed intact, we decided to keep the original ANOVA analyses in the manuscript, especially with regard to preregistration (https://osf.io/pfy7r), where this statistical analysis method was specified. However, we included a brief explanation of the aim of applying clustering the blocks:

“To facilitate data processing and filter out noise, the blocks of ASRT were organized into units of five consecutive blocks (Barnes et al., 2008; Bennett et al., 2007; Nemeth et al., 2010), for which we calculated statistical learning and general skill learning scores.” (pp. 10)

3) “Moreover, the authors have decided to include a Bayesian analysis. However, the results of that analysis are not discussed. For example, in the General skill learning ANOVA a Block x Group interaction was observed in the frequentist analysis with a p-value of 0.04, however, the Bayes factor is at odds with this conclusion and actually suggests the opposite with a BF>1. This would actually cast doubt on the validity of this interaction effect. This part of the analysis is not discussed anywhere, making the BF factor a bit of an afterthought. If they would like to keep this aspect of the analysis, I would suggest that they actually discuss the implications of this addition or leave it out completely.”

Thank you for drawing our attention to this issue. As the interpretation of BF values is detailed in the Statistical analysis section, where BF values were consistent with traditional frequentist statistics we did not consider it necessary to interpret these values separately. We looked through the results and found that in almost all cases the results of the frequentist and Bayesian analysis were consistent. All the results that were deemed important to our conclusions were the same with both approaches. However, we agreed that it is important to highlight the discrepancy between frequentist statistics and BF value in one case although it is not a crucial result and we did not draw conclusions from it. To do so, we added the following sentence to the interpretation of the result in question:

„However, the BFexclusion score of the interaction is above 3, which indicates moderate evidence for the lack of interaction; thus, the interaction is deemed unreliable.” (pp. 13)

-

4) „Lastly, the authors decide to subtract the median reaction time as part of their normalization procedure and dividing by the mean, instead of the more traditional methods of subtracting by the mean. Is there a particular reason for this choice?”

Thank you for this question. The choice of median RTs instead of mean RTs was made because, in the ASRT task, RTs are not normally distributed at the level of individual trials. Thus, using median values could be a better option than using mean values because they can better capture the central tendency of a non-normal distribution and are less sensitive to the long tail of distribution, including outliers. This consideration was particularly important due to online data collection, as the lower experimental control allowed outlying values to occur even more easily.

References

Barnes, K.A., Howard, J.H., Howard, D. V., Gilotty, L., Kenworthy, L., Gaillard, W.D., Vaidya, C.J., 2008. Intact Implicit Learning of Spatial Context and Temporal Sequences in Childhood Autism Spectrum Disorder. Neuropsychology. https://doi.org/10.1037/0894-4105.22.5.563

Bennett, I.J., Howard, J.H., Howard, D. V., 2007. Age-related differences in implicit learning of subtle third-order sequential structure. Journals Gerontol. - Ser. B Psychol. Sci. Soc. Sci. https://doi.org/10.1093/geronb/62.2.P98

Fanuel, L., Pleche, C., Vékony, T., Janacsek, K., Nemeth, D., Quentin, R., 2022. How does the length of short rest periods affect implicit probabilistic learning? Neuroimage: Reports 2, 100078. https://doi.org/10.1016/J.YNIRP.2022.100078

Hallgató, E., Gyori-Dani, D., Pekár, J., Janacsek, K., Nemeth, D., 2013. The differential consolidation of perceptual and motor learning in skill acquisition. Cortex. https://doi.org/10.1016/j.cortex.2012.01.002

Hedenius, M., Persson, J., Alm, P.A., Ullman, M.T., Howard, J.H., Howard, D. V., Jennische, M., 2013. Impaired implicit sequence learning in children with developmental dyslexia. Res. Dev. Disabil. https://doi.org/10.1016/j.ridd.2013.08.014

Kobor, A., Janacsek, K., Takacs, A., Nemeth, D., 2017. Statistical learning leads to persistent memory: Evidence for one-year consolidation. Sci. Rep. https://doi.org/10.1038/s41598-017-00807-3

Nemeth, D., Janacsek, K., 2011. The dynamics of implicit skill consolidation in young and elderly adults. Journals Gerontol. - Ser. B Psychol. Sci. Soc. Sci. https://doi.org/10.1093/geronb/gbq063

Nemeth, D., Janacsek, K., Londe, Z., Ullman, M.T., Howard, D. V., Howard, J.H., 2010. Sleep has no critical role in implicit motor sequence learning in young and old adults. Exp. Brain Res. https://doi.org/10.1007/s00221-009-2024-x

Quentin, R., Fanuel, L., Kiss, M., Vernet, M., Vékony, T., Janacsek, K., Cohen, L.G., Nemeth, D., 2021. Statistical learning occurs during practice while high-order rule learning during rest period. npj Sci. Learn. https://doi.org/10.1038/s41539-021-00093-9

Twilhaar, E.S., de Kieviet, J.F., van Elburg, R.M., Oosterlaan, J., 2019. Implicit Learning Abilities in Adolescents Born Very Preterm. Dev. Neuropsychol. https://doi.org/10.1080/87565641.2019.1620231
