# Supplementary material for: Manipulating the Rapid Consolidation Periods in a Learning Task Affects General Skills More than Statistical Learning and Changes the Dynamics of Learning
Source: eNeuro. 2023 Feb 23;10(2):ENEURO.0228-22.2022. doi: 10.1523/ENEURO.0228-22.2022 (PMC9961365; doi:10.1523/ENEURO.0228-22.2022)
Supplement: Figure 2-2 — The results of general skill learning without age-based exclusion. We have excluded 11 participants from the main analyses to equalize the mean age between groups to ensure that age-related differences have no effect on our results. To test whether the results of general skill learning are biased by exclusions, we run the same ANOVA without exclusions. The results shown in Figure 2 stayed intact. Download Figure 2-2, DOCX file. [file enu-eN-CFN-0228-22-s03.docx]

**Figure 2-2. The results of general skill learning without age-based exclusion.**

| Predictor | *df1* | *df2* | *F* | *p* | *η_p_^2^* | *BF_exclusion_* |
| --- | --- | --- | --- | --- | --- | --- |
| Blocks | 2.76 | 760.79 | 290.75 | <.001^***^ | .51 | <0.001 |
| Group | 2 | 276 | 9.77 | <.001^***^ | .07 | 0.01 |
| Blocks×Group | 8 | 1104 | 2.36 | .04^*^ | .02 | 1.18 |
