# Supplementary figures and images for: Manipulating the Rapid Consolidation Periods in a Learning Task Affects General Skills More than Statistical Learning and Changes the Dynamics of Learning
Source: eNeuro. 2023 Feb 23;10(2):ENEURO.0228-22.2022. doi: 10.1523/ENEURO.0228-22.2022 (PMC9961365; doi:10.1523/ENEURO.0228-22.2022)

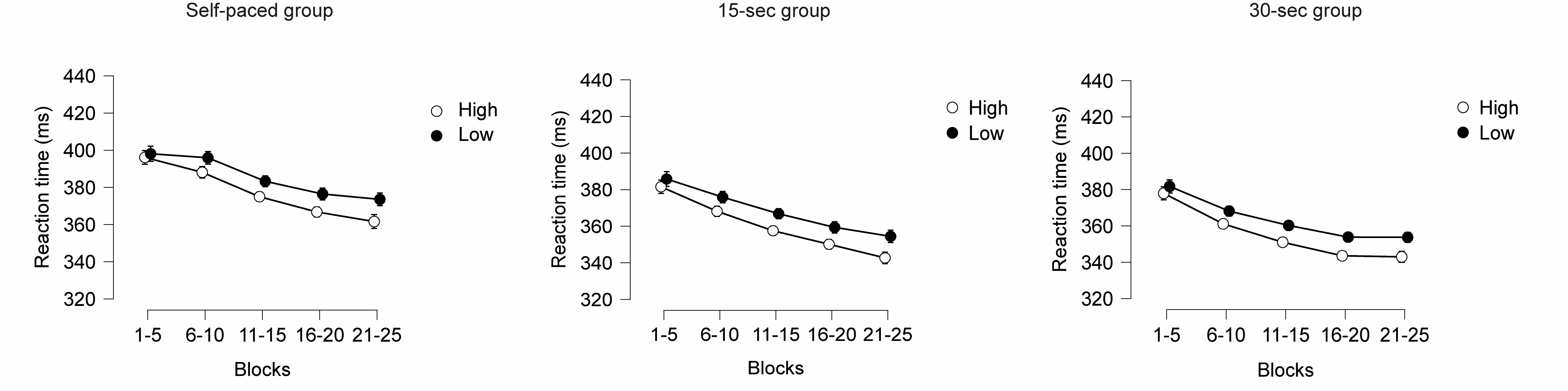

Supplement: Figure 2-3 — Performance of high-probability and low-probability triplets in the three groups. Figure 2 shows the calculated statistical learning scores, while here we depicted the original high-probability (empty circles) and low-probability (filled circles) triplet variables in each group. The y-axes indicate the median RT. The x-axes show the blocks grouped by five. The error bars represent the 95% confidence interval. Download Figure 2-3, TIF file. [file enu-eN-CFN-0228-22-s04.tif]

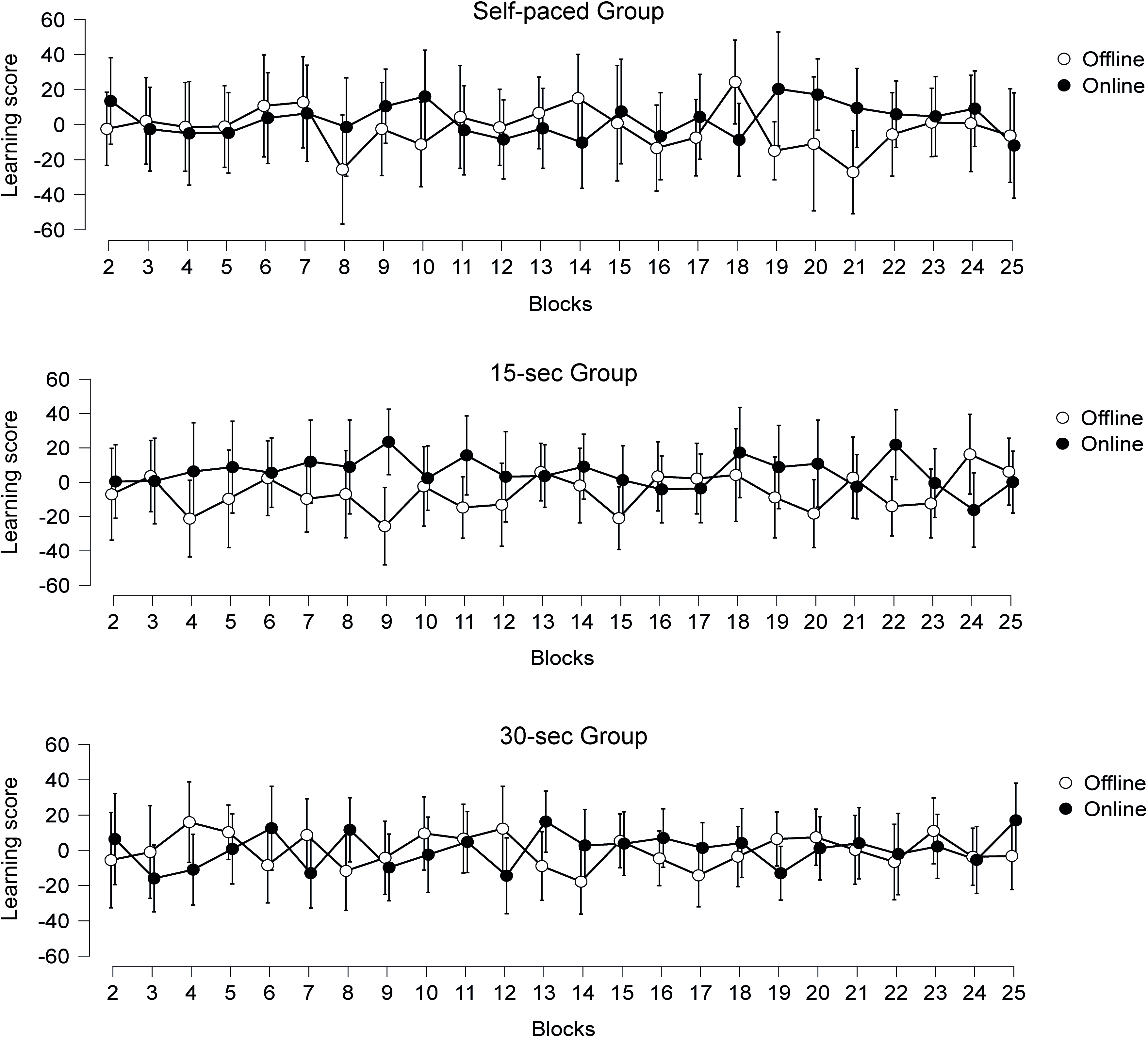

Supplement: Figure 3-3 — Dynamic change of offline and online learning scores across all blocks in each group. The y-axis indicates the mean learning score. The x-axis shows the blocks. The error bars represent the 95% confidence interval. There are only 24 blocks because offline learning score could not be calculated for the first block. It could be seen that the 15 s group is the only one where online learning scores are consistently higher than offline learning scores throughout the task. Download Figure 3-3, TIF file. [file enu-eN-CFN-0228-22-s07.tif]
