# Supplementary material for: Manipulating the Rapid Consolidation Periods in a Learning Task Affects General Skills More than Statistical Learning and Changes the Dynamics of Learning
Source: eNeuro. 2023 Feb 23;10(2):ENEURO.0228-22.2022. doi: 10.1523/ENEURO.0228-22.2022 (PMC9961365; doi:10.1523/ENEURO.0228-22.2022)
Supplement: Figure 3-1 — The results of offline versus online statistical learning without age-based exclusion. We have excluded 11 participants from the main analyses to equalize the mean age between groups to ensure that age-related differences have no effect on our results. To test whether the results of offline-online statistical learning are biased by these exclusions, we run the same ANOVA without exclusions. The results shown in Figure 3 stayed intact. Download Figure 3-1, DOCX file. [file enu-eN-CFN-0228-22-s05.docx]

| Predictor | *df1* | *df2* | *F* | *p* | *η_p_^2^* | *BF_exclusion_* |
| --- | --- | --- | --- | --- | --- | --- |
| Learning Phase | 2 | 276 | 3.51 | .06 | .01 | 0.44 |
| Group | 2 | 276 | 1.09 | .34 | .01 | 45.90 |
| LP×Group | 2 | 276 | 3.17 | .04^*^ | .02 | 0.09 |

**Figure 3-1. The results of offline vs. online statistical learning without age-based exclusion.**
