# Supplementary material for: Manipulating the Rapid Consolidation Periods in a Learning Task Affects General Skills More than Statistical Learning and Changes the Dynamics of Learning
Source: eNeuro. 2023 Feb 23;10(2):ENEURO.0228-22.2022. doi: 10.1523/ENEURO.0228-22.2022 (PMC9961365; doi:10.1523/ENEURO.0228-22.2022)
Supplement: Figure 3-2 — The results of offline versus online general skill learning without age-based exclusion. We have excluded 11 participants from the main analyses to equalize the mean age between groups to ensure that age-related differences have no effect on our results. To test whether the results of offline-online general skill learning are biased by these exclusions, we run the same ANOVA without exclusions. The results shown in Figure 3 stayed intact. Download Figure 3-2, DOCX file. [file enu-eN-CFN-0228-22-s06.docx]

| Predictor | *df1* | *df2* | *F* | *p* | *η_p_^2^* | *BF_exclusion_* |
| --- | --- | --- | --- | --- | --- | --- |
| Learning Phase | 2 | 276 | 645.95 | <.001^***^ | .70 | <0.001 |
| Group | 2 | 276 | 0.17 | .85 | <.001 | 45.35 |
| LP×Group | 2 | 276 | 3.75 | .03^*^ | .03 | 0.03 |

**Figure 3-2. The results of offline vs. online general skill learning without age-based exclusion.**
