# Supplementary material for: Manipulating the Rapid Consolidation Periods in a Learning Task Affects General Skills More than Statistical Learning and Changes the Dynamics of Learning
Source: eNeuro. 2023 Feb 23;10(2):ENEURO.0228-22.2022. doi: 10.1523/ENEURO.0228-22.2022 (PMC9961365; doi:10.1523/ENEURO.0228-22.2022)
Supplement: Figure 4-1 — The distribution of positive and negative offline statistical learning scores in the groups. As offline and online statistical learning scores in the self-paced and 30 s groups were not different from zero at a group level, we have checked whether there is a difference within the groups between the numbers of online and offline learning and forgetting scores. We compared the distribution of those who had high positive (≥5) or high negative (less than or equal to –5) offline learning scores in the three groups. The distribution of those who learned or forgot offline is in balance in the self-paced and the 30 s groups, which could result in no offline learning at the group level. However, in the 15 s group, more participants forgot than learned offline, which resulted in offline forgetting at a group level. Download Figure 4-1, DOCX file. [file enu-eN-CFN-0228-22-s09.docx]

|  | | | | | | | | | |
| --- | --- | --- | --- | --- | --- | --- | --- | --- | --- |
|  | | **Group** | | | | | |  | |
|  | | **Self-paced** | | **15-sec** | | **30-sec** | | **Total** | |
| Learn offline |  | 38 |  | 25 |  | 27 |  | 90 |  |
| Forget offline |  | 31 |  | 49 |  | 34 |  | 114 |  |
| Total |  | 69 |  | 74 |  | 61 |  | 204 |  |
|  | | | | | | | | | |

*Note.* Chi-square test: χ²(2) = 6.57, *p* < 0.05

**Figure 4-1. The distribution of positive and negative offline learning scores in the groups.**
