# Supplementary material for: Manipulating the Rapid Consolidation Periods in a Learning Task Affects General Skills More than Statistical Learning and Changes the Dynamics of Learning
Source: eNeuro. 2023 Feb 23;10(2):ENEURO.0228-22.2022. doi: 10.1523/ENEURO.0228-22.2022 (PMC9961365; doi:10.1523/ENEURO.0228-22.2022)
Supplement: Figure 4-2 — The distribution of positive and negative online learning scores in the groups. Distributions of high positive (≥5) and high negative (less than or equal to –5) learning scores in the three groups were tested for online learning. Similar to offline learning, in the self-paced and the 30 s group, the proportions of those who learned or forgot online are similar, while in the 15 s group, almost twice as many participants learned as forgot online. Download Figure 4-2, DOCX file. [file enu-eN-CFN-0228-22-s10.docx]

|  | | | | | | | | | |
| --- | --- | --- | --- | --- | --- | --- | --- | --- | --- |
|  | | **Group** | | | | | |  | |
|  | | **Self-paced** | | **15-sec** | | **30-sec** | | **Total** | |
| Learn online |  | 34 |  | 50 |  | 34 |  | 118 |  |
| Forget online |  | 38 |  | 25 |  | 26 |  | 89 |  |
| Total |  | 72 |  | 75 |  | 60 |  | 207 |  |
|  | | | | | | | | | |

*Note.* Chi-square test: χ²(2) = 5.67, *p* = 0.06

**Figure 4-2. The distribution of positive and negative online learning scores in the groups.**
