# Supplementary material for: Manipulating the Rapid Consolidation Periods in a Learning Task Affects General Skills More than Statistical Learning and Changes the Dynamics of Learning
Source: eNeuro. 2023 Feb 23;10(2):ENEURO.0228-22.2022. doi: 10.1523/ENEURO.0228-22.2022 (PMC9961365; doi:10.1523/ENEURO.0228-22.2022)
Supplement: Figure 3-4 — The results of ANOVAs for offline and online changes across all blocks in the separate groups. To see how offline and online statistical learning dynamically change across all blocks in the separate groups, we conducted three ANOVAs with the offline and online statistical learning scores of all blocks separately for each group. The results of the three ANOVAs confirm no effect of time on offline and online learning, as the main effect of Blocks was not significant in either group. Download Figure 3-4, DOCX file. [file enu-eN-CFN-0228-22-s08.docx]

| Group | Predictor | *df1* | *df2* | *F* | *p* | *η_p_^2^* | *BF_exclusion_* |
| --- | --- | --- | --- | --- | --- | --- | --- |
| **Self-paced** | Blocks | 10.56 | 401.26 | 0.85 | .59 | .02 | <0.001 |
|  | Learning Phase | 1 | 38 | 0.88 | .35 | .02 | 11.44 |
|  | Blocks×LP | 12.82 | 487.30 | 0.69 | .77 | .02 | <0.001 |
| **15-sec** | Blocks | 9.98 | 468.87 | 0.46 | .92 | .01 | <0.001 |
|  | Learning Phase | 1 | 47 | 7.57 | <.01^**^ | .14 | 0.05 |
|  | Blocks×LP | 13.71 | 644.47 | 0.97 | .48 | .02 | <0.001 |
| **30-sec** | Blocks | 10.82 | 486.66 | 0.77 | .67 | .02 | <0.001 |
|  | Learning Phase | 1 | 45 | 0.01 | .94 | .001 | 31.11 |
|  | Blocks×LP | 13.51 | 607.93 | 1.02 | .43 | .02 | <0.001 |

**Figure 3-4. The results of ANOVAs for offline and online changes across all blocks in the separate groups.**
